# Butyrate Supplementation Improves Intestinal Health and Growth Performance in Livestock: A Review

**DOI:** 10.3390/biom15010085

**Published:** 2025-01-08

**Authors:** Wenting Chen, Qingshan Ma, Yan Li, Lin Wei, Zhenwei Zhang, Adnan Khan, Muhammad Zahoor Khan, Changfa Wang

**Affiliations:** 1 School of Agricultural Science and Engineering, Liaocheng University, Liaocheng 252000, China; 2 Animal Genomics Laboratory, School of Agriculture and Food Science, University College Dublin, D04 V1W8 Dublin, Ireland; adnan.khan@ucd.ie

**Keywords:** butyrate, livestock, microbiota, intestinal health, inflammation, antioxidants, growth performance

## Abstract

Butyrate supplementation has gained considerable attention for its potential benefits in livestock, particularly concerning intestinal health and growth performance. This review synthesizes recent research on the diverse roles of butyrate, across various livestock species. As a short-chain fatty acid, butyrate is known for enhancing intestinal development, improving immune function, and modulating microbial diversity. Studies indicate that butyrate supports gut barrier integrity, reduces inflammation, and optimizes feed efficiency, especially during the critical weaning and post-weaning periods in calves, piglets, and lambs. Supplementation with butyrate in livestock has been shown to increase average daily gain (ADG), improve gut microbiota balance, promote growth, enhance gut health, boost antioxidant capacity, and reduce diarrhea. Additionally, butyrate plays a role in the epigenetic regulation of gene expression through histone acetylation, influencing tissue development and immune modulation. Its anti-inflammatory and antioxidant effects have been demonstrated across various species, positioning butyrate as a potential therapeutic agent in animal nutrition. This review suggests that optimizing butyrate supplementation strategies to meet the specific needs of each species may yield additional benefits, establishing butyrate as an important dietary additive for enhancing growth performance and health in livestock.

## 1. Introduction

Butyrate plays a critical role in animal health, serving not only as an important energy source for colonocytes but also as a cellular mediator that regulates various functions in intestinal cells and other tissues [1,2]. Additionally, it is a key supplement that effectively promotes intestinal development, enhances overall health, and improves growth performance [3,4,5,6,7]. Recent studies have emphasized its role in strengthening the integrity of the gut barrier and supporting a balanced gut microbiota. For example, sodium butyrate supplementation has been shown to significantly increase the average daily gain (ADG) and enhance microbiota diversity, notably impacting *Firmicutes*, *Bacteroidetes*, and *Lactobacillus*, which are crucial for calf development [8,9,10]. Moreover, butyrate supplementation supports improvements in gastrointestinal structures, such as the development of rumen papillae and duodenal villi, which are essential for nutrient absorption and overall gut health [11]. Besides these structural benefits, continuous supplementation of butyrate can reduce diarrhea incidence, enhance calf health, and improve postnatal growth and immune responses [11,12].

One of the most notable advantages of butyrate supplementation is its ability to promote healthy gut microbiota, which are vital for optimal growth and health. Specifically, butyrate increases microbial diversity and supports the growth of beneficial bacteria such as *Firmicutes*, *Bacteroides*, *Prevotella*, and *Ruminococcus*, while simultaneously reducing harmful pathogens like *Proteobacteria* and *Escherichia/Shigella* [13,14]. This beneficial modulation of the gut microbiota is particularly significant during early intestinal development in animals, when microbial diversity is critical for establishing a robust digestive system.

In addition to modulating the gut microbiota, butyrate also plays a crucial role in immune regulation and inflammation control. It reduces the production of pro-inflammatory cytokines, inhibits the activation of nuclear factor kappa B (NF-κB), and protects the integrity of tight junctions in epithelial cells [15]. Notably, sodium butyrate supplementation has been linked to decreased levels of inflammatory markers such as interleukin-1β (IL-1β), IL-6, and tumor necrosis factor-α (TNF-α), leading to improved immune responses and reduced stress during critical growth phases [16,17]. Furthermore, these immune-regulatory effects extend to enhancing resistance to infections, as demonstrated by studies where butyrate improved host defenses against *Mycobacterium bovis*, the causative agent of bovine tuberculosis [18].

At the cellular level, butyrate influences gene expression through epigenetic mechanisms, particularly by inhibiting histone deacetylases (HDACs), which results in increased histone acetylation [19]. This process promotes the transcription of anti-inflammatory genes while suppressing the expression of pro-inflammatory cytokines, thereby enhancing anti-inflammatory responses [20,21]. Additionally, butyrate regulates key signaling pathways, such as AMP-activated protein kinase (AMPK) and mitogen-activated protein kinase (MAPK), which are essential for tissue development and metabolic regulation [15,22]. These epigenetic effects are particularly beneficial for improving growth performance and supporting animal health during stressful periods such as weaning.

**Figure 1:**
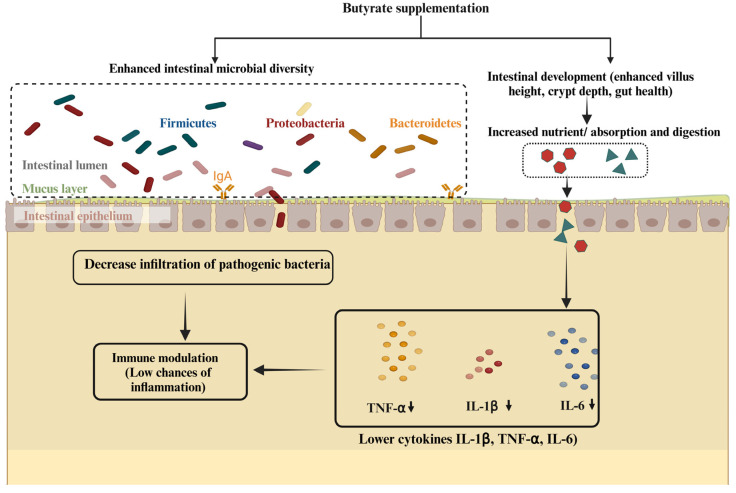
Butyrate’s role in intestinal development, microbiota balance, immune response, and promotion of antiinflammation. Butyrate plays a pivotal role in maintaining microbiota balance, promoting intestinal development, and enhancing growth performance. It supports anti-inflammatory mechanisms by reducing levels of pro-inflammatory cytokines such as IL-6, IL-1β, and TNF-α. Additionally, butyrate contributes to immune regulation by modulating immune cell function, thereby supporting intestinal health and sustaining overall immune homeostasis.

This review aims to synthesize and assess the current research on the effects of butyrate supplementation on intestinal health and growth performance in livestock, including calves, piglets, sheep, and goats. As a short-chain fatty acid, butyrate plays a pivotal role in enhancing gut barrier function, modulating immunity, maintaining microbial balance, and influencing epigenetic regulation. This comprehensive overview highlights the multifaceted benefits of butyrate, including growth promotion, increased microbial diversity, inflammation control, and improved cellular functions (Figure 1). It offers valuable insights for researchers and industry professionals aiming to optimize livestock health and productivity.

## 2. Methodology for Literature Search

This review synthesized data from published articles ranging from 2017 to the present, along with five additional articles published between 2011 and 2016, to ensure a comprehensive analysis of the content. The literature search employed the following keywords: “butyrate”, “intestinal health”, “growth”, “animal production performance”, “inflammation”, “immunity”, “intestinal microbiota”, “sodium butyrate”, “tributyrin”, “livestock”, “cows”, “pigs”, “sheep”, and “goats”. We exclusively considered articles published in peer-reviewed journals indexed in the Science Citation Index (SCI) and written in the English language. Additionally, we excluded data from books, book chapters, conference papers, and unpublished materials to maintain the rigor and relevance of the review. Finally, all the figures used in this study were created using https://BioRender.com.

## 3. Butyrate Role in Livestock Intestinal Health and Growth Performance

Butyrate is naturally produced in the large intestine and the foregut of ruminants through bacterial fermentation, a process primarily mediated by the enzyme butyryl-CoA/acetate-CoA transferase. Importantly, a balanced diet rich in fermentable fiber and non-structural carbohydrates can optimize butyrate production. Butyrate plays a crucial role in livestock nutrition, with numerous studies consistently demonstrating its significant positive effects on growth, intestinal health, and modulation of the gut microbiota [23,24,25,26]. Notably, the multifunctional properties of butyrate extend beyond its role in growth promotion; it also enhances intestinal integrity and shows promise as a treatment for various gastrointestinal disorders [27,28,29,30,31,32,33]. Furthermore, this short-chain fatty acid regulates critical gastrointestinal functions by modulating epithelial cell proliferation, apoptosis, and differentiation in response to microbial growth and signaling mechanisms [10,34]. In the following sections, we have discussed the essential role of butyrate in promoting livestock health, with a focus on its anti-inflammatory, antioxidant, and immune-regulating properties, as well as its impact on growth performance, including microbiota balance, daily weight gain, and enhanced intestinal development.

### 3.1. Butyrate’s Role in Calf Intestinal Health and Growth Promotion

Butyrate, as a key feed additive, plays a significant role in promoting intestinal health and growth in calves, particularly during the vulnerable weaning and post-weaning periods. Recent studies have shown that butyrate has multiple effects, including supporting intestinal development, regulating immune responses, maintaining gut barrier integrity, and promoting gut microbiota balance in calves [8,35,36]. The supplementation with 8.78 g of sodium butyrate per day significantly increased ADG and positively affected microbial diversity and composition, notably enhancing the populations of *Lactobacillus*, *Ruminococcus*, *Romboutsia*, and *Erysipelotrichaceae_UCG-003* [9]. Furthermore, a quadratic pattern was observed in the improvements in growth performance and gut health, suggesting that adjusting the sodium butyrate dosage provides targeted benefits [9]. Consistently, research on supplementing 45 g/day of sodium butyrate showed significant improvements in bacterial diversity. Further, it has been shown to promote the proliferation of beneficial bacterial genera, including *Firmicutes*, *Bacteroides*, *Prevotella*, and *Ruminococcus*, while simultaneously decreasing the abundance of potentially pathogenic bacteria such as *Proteobacteria* and *Escherichia-Shigella*. These findings further confirm the critical role of sodium butyrate in promoting a balanced and robust microbial environment during the early stages of calf development [13]. Notably, the effects of sodium butyrate are not limited to microbiota modulation. A study investigating the continuous addition of sodium butyrate to the liquid diet of dairy calves demonstrated a significant reduction in the incidence of diarrhea and overall morbidity. Furthermore, calves receiving 4 g of sodium butyrate per day showed improvements in growth, gastrointestinal development, and immune responses, underscoring the importance of butyrate in enhancing the structural and functional aspects of the digestive system during the critical post-weaning period [11]. Similarly, another study examined the impact of sodium butyrate as a milk replacer additive in Holstein’s calves. Twenty-four calves were divided into three groups receiving 0, 5, or 10 g of sodium butyrate per day for 49 days. Their results indicated that sodium butyrate supplementation improved feed conversion ratios and ADG, with the 5 g per day group showing the most pronounced benefits. Additionally, sodium butyrate increased total blood protein, enhanced rumen bacterial populations, and lowered ruminal pH, ultimately promoting rumen microbial development and energy metabolism [24]. In a parallel study, researchers explored the effects of sodium butyrate on the transcriptomic pathways of the gastrointestinal epithelium and the rumen microbiome in calves fed a high-fiber diet [3]. Their results showed that sodium butyrate supplementation improved ADG, enhanced jejunum and rumen papillae development, and downregulated inflammatory pathways such as IL-17 and NF-κB. Additionally, sodium butyrate increased the populations of beneficial bacteria, including *Bacillus subtilis* and *Eubacterium limosum*, further emphasizing its role in promoting calf health and nutrition [3].

Similarly, another study investigated the effects of sodium butyrate on pre-weaning calves, focusing on growth performance, diarrhea incidence, gastrointestinal tract (GIT) development, and tight junction protein expression. Sodium butyrate treatment effectively reduced diarrhea rates and increased rumen weight and papillae surface area. Additionally, sodium butyrate increased mucosal thickness in the abomasum and duodenum, improved ileal villus height and villus height-to-crypt depth ratios, and upregulated claudin-1 expression in the rumen, which was negatively correlated with diarrhea incidence. Furthermore, adding 4 g/day of sodium butyrate to milk replacer from day 4 to day 60 significantly affected starter feed intake, total dry matter intake, and hematological parameters such as glucose, urea, insulin-like growth factor-1 (IGF-1), and differential blood cell counts [24,30]. Furthermore, extending these findings, the research evaluated the impact of sodium butyrate supplementation on glucagon-like peptide (GLP)-1 and GLP-2 levels in beef cattle during late pregnancy and early postpartum periods. Twelve Japanese black cows were divided into a treatment group receiving sodium butyrate supplementation and a control group [37]. They further showed that the treatment group had significantly increased plasma total cholesterol and GLP-1 concentrations three days after calving. Notably, GLP-1 concentrations were higher in colostrum compared to plasma, although the treatment did not affect the composition of colostrum and transition milk [37]. In another significant study, the effects of sodium butyrate on pancreatic development in dairy calves over a 70-day period were examined [31]. Fourteen male Holstein calves were divided into two groups and fed either standard milk or milk supplemented with sodium butyrate. The results revealed that sodium butyrate significantly stimulated pancreatic growth, as evidenced by increased organ size, enhanced protein content, and elevated cellular proliferation. Notably, exocrine pancreatic function showed marked improvements, including higher enzyme activity and upregulated gene expression. Endocrine benefits were also observed, with increased insulin secretion and β-cell proliferation. Proteomic analysis identified G protein subunit alpha-15 (GNA15) as a key regulator of these processes, underscoring the role of sodium butyrate in promoting both exocrine and endocrine pancreatic functions in calves [31].

Effects of encapsulated butyric acid and zinc (BZ) with varying doses have been shown to be associated with growth, rumen morphology, and small intestine histology of feedlot steers [38]. Furthermore, it has been revealed that BZ supplementation (2 g BZ/kg dietary dry matter) improved dressed yield and growth performance during the receiving period and reduced liver abscess incidence. The researchers proposed that BZ supplementation could enhance early growth and decrease liver abscess incidence, though further research is needed to confirm these findings [38]. Additionally, magnesium butyrate (MgB) supplementation at a rate of 105 g/cow/day significantly increased colostrum yield and immunoglobulin G (IgG) levels, enhanced milk yield during the first week of lactation, and improved body condition scores from 3 to 9 weeks postpartum [12]. Interestingly, MgB also delayed the decline in milk production, improved milk synthesis efficiency, increased milk fat content, and reduced pro-inflammatory cytokines and somatic cell counts by inhibiting bacterial cell wall components [39]. Furthermore, the effects of sodium butyrate and sodium acetate supplementation on diarrheic yak calves in Tibet have been evaluated [40]. They used nineteen calves (divided into control and treatment groups), with the treatment group receiving sodium butyrate (10 g/kg) and sodium acetate (5 g/kg) for 28 days. Notably, their analysis showed increased short-chain fatty acids (SCFAs) and beneficial shifts in gut microbiota composition in the supplemented group. Moreover, the supplemented group experienced reduced diarrhea incidence, improved weight gain, enhanced total antioxidant capacity (T-AOC), superoxide dismutase (SOD), and glutathione peroxidase (GSH-Px) levels, as well as reduced inflammatory responses, including decreases in TNF-α and IL-1β levels [40]. Building upon these findings, a notable study explored the effects of sodium butyrate supplementation in calf starters with varying starch levels. Conducted over a 65-day period, the study focused on the inflammatory response and growth performance of post-weaned dairy calves [30]. Remarkably, sodium butyrate supplementation (5 g/kg of dietary dry matter) significantly increased ADG, particularly in calves consuming a low-starch starter (270 g/kg of dietary dry matter). Additionally, key inflammatory markers, such as toll-like receptor 4 (TLR4), nuclear factor kappa B (p65), and several pro-inflammatory cytokines were affected by dietary intervention, underscoring the importance of tailored sodium butyrate strategies in optimizing post-weaning calf growth and health [30]. This perspective is consistent with numerous studies demonstrating the challenges faced during the weaning process, which not only requires rumen development [41] but also the regulation of inflammatory responses [42,43]. In this context, current research increasingly focuses on identifying key inflammatory biomarkers that can predict disease susceptibility and growth outcomes in calves [44]. Complementing these findings, another study showed that supplementing high-starch calf starters (365 g of starch/kg of starter) with rumen-protected butyrate (10 g/kg butyrate/starter) during the critical two-week weaning transition increased starter intake, which may reflect a reduction in inflammation and stress during this period [16]. Furthermore, research has explored the protective effects of sodium butyrate against lipopolysaccharide (LPS)-induced inflammation in bovine rumen epithelial cells (BRECs), focusing on the role of G protein-coupled receptor 41 (GPR41) activation [45]. Pretreatment with sodium butyrate successfully reduced the expression of pro-inflammatory genes and increased the levels of tight junction proteins in LPS-stimulated BRECs. Additionally, sodium butyrate inhibited the activation of NF-κB and IκBα, decreased the expression of apoptosis markers such as Bax, caspase 3, and caspase 9, and favorably altered cell cycle distribution. Sodium butyrate also promoted the expression of genes involved in volatile fatty acid metabolism (*ACAT1* and *BDH1*) and upregulated *GPR41* expression. Notably, the protective effects of short-chain fatty acids, including sodium butyrate, were not observed in GPR41 knockdown BRECs, indicating that GPR41 activation is crucial for sodium butyrate’s anti-inflammatory effects [46].

The antioxidant potential of butyrate has also been documented. Consistently, a study investigated the protective effects of sodium butyrate against LPS-induced mastitis in bovine mammary epithelial cells (BMECs) [47]. Based on their findings, sodium butyrate significantly reduced LPS-induced cell death in a concentration-dependent manner, lowered oxidative stress (as indicated by reduced intracellular reactive oxygen species (ROS) and malondialdehyde (MDA) levels), and enhanced the activity of antioxidant enzymes including SOD, catalase (CAT), and GPX. Moreover, it suppressed inflammatory markers (IL-6, IL-1β, TNF-α) and apoptosis-related proteins (caspases, Bax, Bcl-2), suggesting that it regulates oxidative stress, inflammation, and apoptosis in BMECs through the NF-κB and caspase/Bax pathways [47]. The research also showed that sodium butyrate exhibited anti-inflammatory effects on bovine embryo tracheal cells (EBTr), reversing LPS-induced inflammatory responses by reducing the expression of TLR4, phosphorylated NF-κB, IκBα, and IL1α, and inhibiting the nuclear translocation of NF-κB p65 through modulation of the TLR4 and NF-κB signaling pathways [48]. Interestingly, sodium butyrate also played a key role in inhibiting neutrophil extracellular trap (NET) formation induced by Staphylococcus aureus. Notably, sodium butyrate reduced the expression of NET markers, such as DNA, histones, myeloperoxidase, and neutrophil elastase [49]. Additionally, sodium butyrate activated the mammalian target of the rapamycin (mTOR) pathway, providing further mechanistic insights into its NET-suppressing properties [49]. Sodium butyrate also exhibited significant anticoccidial potential by reducing the viability of *Eimeria bovis* sporozoites and protecting Madin-Darby bovine kidney (MDBK) cells from damage, as evidenced by decreased lactate dehydrogenase (LDH) release. Furthermore, sodium butyrate supplementation (45 g/day) improved growth rates, ADG, feed conversion efficiency, and serum antioxidant markers, including increased GSH-Px activity and decreased MDA levels [50,51,52].

Tributyrin is a triglyceride formed by three butyrate molecules bound to a glycerol backbone. When ingested or administered, tributyrin is broken down by digestive enzymes (such as lipases), releasing butyrate. This process makes tributyrin an effective delivery form of butyrate, particularly in situations where direct butyrate supplementation may be difficult or inefficient. As a precursor of butyric acid, tributyrin has demonstrated significant benefits in reducing diarrhea and enhancing intestinal barrier function in calves. Studies have reported increased ADG, higher weaning weights, and reduced oxidative stress, evidenced by lower ROS and MDA levels, along with higher SOD and serum immunoglobulin M (IgM) levels. Additionally, tributyrin supplementation has been associated with an increase in beneficial gut bacteria, such as *Rikenellaceae*, *Bacteroides*, and *Ruminococcaceae*, as well as improvements in ileal morphology, plasma GLP-2 concentration, and post-weaning body weight [53,54,55,56,57]. Furthermore, tributyrin supplementation significantly reduced diarrhea frequency in calves, correlating with improved intestinal development and barrier function, as indicated by increased villus height and claudin-4 expression [53]. For example, as a derivative of butyrate, tributyrin effectively regulated inflammation, specifically IL-1β, during the pre-weaning phase, providing further evidence of butyrate’s essential role in managing early-life inflammation [53]. Consistently, another study reported that supplementing milk with 2 g/L tributyrin significantly reduced diarrhea frequency between days 29 and 56, along with notable reductions in the expression of IL-1β, TLR2, and serum amyloid A, while simultaneously increasing claudin-4 and GPR41 levels in the jejunum and ileum. Furthermore, tributyrin supplementation promoted intestinal development, as shown by increased villus height and improved villus height-to-crypt depth ratios in the jejunum. Although no significant changes were observed in microbiota abundance, tributyrin remarkably increased the presence of beneficial SCFA-producing bacteria such as *Ruminococcaceae*, *Lachnospiraceae*, *Prevotella*, and *Rikenellaceae*, which correlated negatively with inflammatory gene expression and positively with intestinal barrier function and morphology [53].

**Figure 2:**
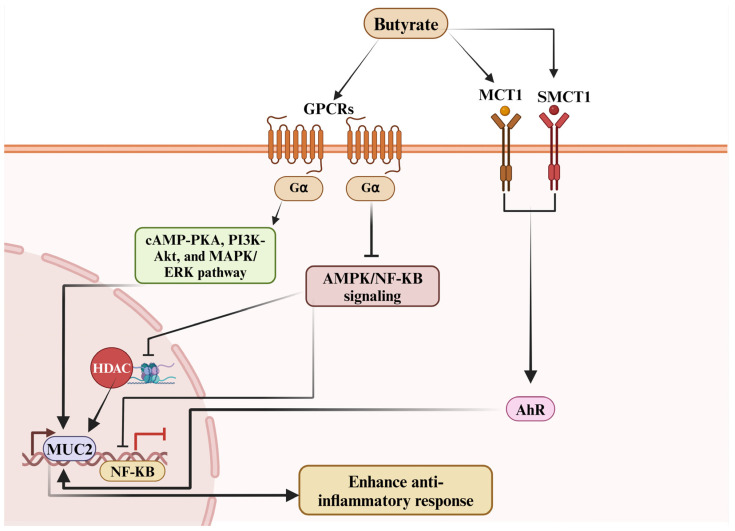
Butyrate’s role in promoting anti-inflammatory response. Butyrate via monocarboxylate transporter 1 (MCT1) and sodium-coupled monocarboxylate transporter 1 (SMCT1) activate aryl hydrocarbon receptor (AHR) to promote anti-inflammatory response.

Butyrate modulates gene expression by inhibiting HDACs, leading to increased histone acetylation, which enhances anti-inflammatory gene transcription and suppresses pro-inflammatory cytokines (Figure 2). A study demonstrated that sodium butyrate prevents LPS-induced inflammation in bovine mammary epithelial cells (MAC-T) by reducing apoptosis, lowering pro-inflammatory cytokines (TNF-α, IL6, IL-1β) in a dose-dependent manner, and preserving histone H3 acetylation. Sodium butyrate also inhibited NF-κB signaling, further supporting its potential as a protective agent against mastitis-related pathogens [58]. Additionally, sodium butyrate has shown therapeutic potential in enhancing host defenses against *Mycobacterium bovis*, the causative agent of bovine tuberculosis. In in vitro studies, sodium butyrate upregulated the expression of the antimicrobial peptide cathelicidin (LL37) in infected cells, while downregulating HDACs and inhibiting NF-κB signaling pathways. Furthermore, in in vivo mouse models, sodium butyrate reduced the production of inflammatory cytokines and enhanced resistance to *M. bovis* infection [18]. In a related study, the effects of butyrate on inflammation and tight junction (TJ) integrity were explored in BMECs [15]. Butyrate treatment significantly lowered HDAC3 levels, inhibited NF-κB activation, and reduced inflammatory cytokine production in response to γ-D-glutamyl-meso-diaminopimelic acid (iE-DAP) stimulation. Additionally, butyrate suppressed the expression of myosin light chain kinase (MLCK), increased tight junction protein levels, and improved epithelial barrier function. Importantly, the overexpression of HDAC3 counteracted these protective effects, demonstrating that butyrate alleviates inflammatory responses and maintains TJ integrity through an HDAC3-dependent mechanism [15]. Building on this, another study found that HDAC activity increased linearly with higher doses of LPS, accompanied by a significant reduction in histone H3 acetylation levels [22]. Beyond its well-established gastrointestinal benefits, sodium butyrate also regulates cellular function and tissue development within the rumen through epigenetic mechanisms. A recent study revealed that butyrate regulates gene expression via genome-wide H3K27ac acetylation, profoundly affecting pathways such as AMPK and MAPK signaling, and further underscoring its roles in cholesterol metabolism, cell adhesion, and overall cellular function in bovine rumen epithelial cells [19]. These findings provide deep insights into the epigenetic regulation of inflammatory responses, particularly highlighting the role of HDACs in modulating histone acetylation and inflammation. Overall, research on sodium butyrate emphasizes its multifaceted role in promoting calf health and development. Studies have shown that sodium butyrate enhances intestinal growth, modulates immune responses, improves microbial diversity, and reduces inflammation, collectively contributing to improved growth performance, feed efficiency, and reduced gastrointestinal disturbances such as diarrhea during critical weaning and post-weaning periods in calves, as summarized in Table 1.

### 3.2. The Role of Butyrate in Piglet Intestinal Health and Growth Promotion

Butyrate plays a crucial role in promoting intestinal health and supporting piglet growth, particularly during the vulnerable post-weaning period, as widely documented in the literature. Chemically protected sodium butyrate has demonstrated significant effects in enhancing gut health, especially in weaned piglets. Notably, supplementation with a high dose of sodium butyrate (2000 mg/kg) led to an 18.5% improvement in ADG, while a lower dose (1000 mg/kg) still achieved a 6.9% increase [69]. Beyond promoting growth, high doses of sodium butyrate significantly upregulated anti-inflammatory cytokines (e.g., IL-10) and key antioxidant markers, including Keap1, Nrf-2, CAT, SOD, and GSH-Px. Additionally, this treatment promoted the proliferation of beneficial SCFA-producing bacteria, suggesting a multifaceted role in enhancing gut health through the modulation of microbiota composition [69]. Furthermore, butyrate serves as a vital energy source for colonocytes and helps alleviate stress in weaned piglets, although the precise mechanisms remain incompletely understood [70]. In the LPS-induced inflammatory model, butyrate supplementation improved gut morphology and reduced inflammation, evidenced by increased villus height and villus height-to-crypt depth ratio, while lowering inflammatory markers such as NF-κB and IL-1β, thereby contributing to intestinal integrity. Additionally, it altered the microbial composition, increasing beneficial bacteria while reducing harmful ones, indicating broad protective effects on gut health during stress and infection [70]. Similarly, adding sodium butyrate to low-protein diets improved the expression of antioxidant and anti-inflammatory genes, further demonstrating the compound’s capability to balance immune responses and oxidative stress [71].

In line with these findings, other studies have shown that butyrate is essential during critical developmental stages such as weaning, where it helps maintain jejunal homeostasis and supports intestinal integrity. Supplementation with sodium butyrate (0.45 g/kg) has been proven to suppress inflammatory mediators such as IL-1β, IL-6, and TNF-α while enhancing intestinal barrier function [72]. Moreover, another form of butyrate, tributyrin, effectively reduced diarrhea incidence and improved gut morphology in nursery piglets [73].

Recent research has further expanded on butyrate’s role beyond gut health, highlighting its potential to reduce liver injury caused by mycotoxins such as deoxynivalenol (DON). Sodium butyrate, through the modulation of oxidative stress pathways and histone acetylation mechanisms, successfully reversed liver damage and inflammation associated with DON exposure, underscoring its therapeutic potential [74]. This finding aligns with previous studies that repeatedly emphasize the ability of sodium butyrate to enhance growth performance and support immune function in piglets [75]. They reported that sodium butyrate significantly improved ADG and feed conversion efficiency, increased ileal villus height, and reduced the number of Peyer’s patches in the ileum. Sodium butyrate treatment also upregulated the expression of several key genes, including nutrient transporters like sodium/glucose cotransporter 1 (*SGLT1*) and monocarboxylate transporter 1 (*MCT1*), tight junction proteins (*OCL* and zonula occludens-1), and antioxidants (*GPX* and *SOD*), which are critical for maintaining intestinal integrity and function [75]. Furthermore, sodium butyrate supplementation in low-protein diets over a four-week period significantly improved gut microbiota composition and markedly reduced inflammatory markers such as IL-8, TLR4, and IKKα in both plasma and colonic mucosa. Moreover, sodium butyrate enhanced antioxidant activity by upregulating GSH-Px expression and increasing Nrf2 activity, thereby effectively mitigating oxidative stress [71].

Additionally, enterotoxigenic *Escherichia coli* (ETEC) strains, including ETEC F4 and ETEC F18, are known to be major contributors to post-weaning diarrhea in piglets. Consistently, Kovanda et al. [76] found that butyrate treatment significantly alleviated diarrhea in piglets co-infected with ETEC F4 and ETEC F18 by reducing neutrophil infiltration and lowering serum TNF-α levels. Similarly, López-Colom et al. [77] highlighted the protective effects of medium-chain fatty acid-protected butyrate in maintaining intestinal health in pigs challenged with ETEC F4. Specifically, butyrate acts as a primary energy source for colonocytes, enhances intestinal barrier integrity, and exhibits strong anti-inflammatory properties, thereby mitigating damage caused by infections. By preserving tight junctions between intestinal cells, butyrate reduces gut permeability, prevents bacterial toxin translocation, and modulates immune responses to reduce inflammation and promote intestinal healing [77].

A study reported that 0.5% glyceryl butyrate supplementation in piglets exhibited significantly lower levels of pro-inflammatory cytokines (IL-1β, IL-6, and TNF-α) in the jejunum and ileum compared to controls [78]. Glyceryl butyrate supplementation also enhanced the expression of porcine beta-defensins (pBDs) and tight junction-associated proteins (Claudin 1, Occludin, and ZO-1), thereby improving gut barrier function. These improvements were attributed to the reduced activation of the NF-κB/MAPK signaling pathways and promoted the growth of beneficial gut microbiota, particularly Lactobacillus species [78]. The dietary supplementation of sows with sodium butyrate, medium-chain fatty acids, and omega-3 polyunsaturated fatty acids has shown a profound positive impact on both reproductive performance and the intestinal health of their piglets [79]. This supplementation regimen resulted in shorter weaning-to-estrus intervals, improved oxidative status, reduced diarrhea incidence, and enhanced colostrum quality [79]. Additionally, Zhong et al. [80] emphasized the critical role of microbial-derived butyrate in regulating cell apoptosis and proliferation, as well as modulating immune responses in the jejunum during the crucial first three weeks post-weaning. Notably, during the first week, a reduction in butyrate production, driven by an increase in specific bacterial families (*Erysipelotrichaceae* and *Lachnospiraceae*), led to inflammation, cell apoptosis, and impaired intestinal development. However, by the second week, an increase in other beneficial bacterial families (*Lactobacillaceae* and *Ruminococcaceae*) promoted butyrate production, which subsequently reduced inflammation, stimulated cell proliferation, and supported intestinal health and development [80]. In summary, these findings underscore the pivotal role of sodium butyrate in promoting growth, enhancing gut health, and alleviating inflammation and oxidative stress, particularly during the vulnerable post-weaning period for piglets, as presented in Table 2.

### 3.3. The Role of Butyrate in Intestinal Health and Growth Promotion in Sheep and Goats

The significant role of butyrate in enhancing intestinal health and promoting growth performance in sheep and goats has been widely documented. Consistent with these findings, supplementation with sodium butyrate (3 g/kg of starter feed for 73 days) significantly improved starter feed intake and increased weaning weight in lambs. The combination of forage and sodium butyrate resulted in positive synergistic effects on lamb weight gain, wither height, and body barrel size [90]. Sodium butyrate also increased the concentrations of SCFA and butyrate, while forage increased ruminal pH and acetate levels, but decreased butyrate and propionate concentrations [90]. A study reported that sodium butyrate supplementation (36 g/kg of dry matter for 14 days) not only increased the final body weight but also raised the butyrate concentrations in both reticuloruminal fluid and abomasal digesta [91]. Notably, significant structural improvements were observed in the omasum and abomasum, including increased epithelial thickness and enhanced hydrolytic activity in the rumen [91,92]. Similarly, Sun et al. [93] reported that administering sodium butyrate (1.8 mL/kg body weight) from days 7 to 35 significantly increased the average daily gain, improved jejunal villus height, and enhanced the villus-to-crypt ratio in the ileum of lambs. Moreover, the treatment promoted the expression of intestinal barrier proteins (Claudin-1 and Occludin) and modulated the gut microbiota, reducing harmful bacteria (e.g., *Clostridia_UCG-014* and *Romboutsia*) while increasing beneficial bacteria (e.g., *Succiniclasticum*) [93]. Furthermore, Świerk et al. [94] demonstrated that sodium butyrate supplementation, combined with higher concentrate intake, affected epithelial thickness in specific rumen regions and upregulated the expression of genes and proteins related to rumen barrier function, such as MCT1 and Claudin-4 [95,96]. Beyond intestinal health, butyrate supplementation also exhibits broader physiological effects. For instance, Mohamed et al. [97] reported that feeding lambs with a milk replacer containing sodium butyrate significantly increased total and daily weight gain, elevated testosterone levels, and improved testicular development. Additionally, the combination of sodium butyrate and milk replacer effectively reduced oxidative stress markers and enhanced antioxidant enzyme activity [97]. Another study investigated the application of the butyrate-producing strain *Clostridium beijerinckii R8*, isolated from sheep rumen, in goat kids [98]. Remarkably, the use of this strain significantly reduced diarrhea incidence and fecal scores, while promoting intestinal health by inhibiting inflammation, enhancing antioxidant capacity, and modulating the gut microbiota. Furthermore, it strengthened the intestinal barrier and improved jejunal morphology, offering a comprehensive approach to enhancing gut health in goat kids [98]. Consistently, Zhao et al. [99] reported that supplementation with coated sodium butyrate (3 g/kg feed for 21 days) in lambs significantly improved growth traits such as ADG and average daily feed intake (ADFI), primarily by promoting the colonic microbiota. This was accompanied by improvements in antioxidant markers, including increased levels of T-AOC, SOD, GSH-Px, and catalase (CAT), while MDA levels were reduced [99]. Furthermore, another study indicated that infection with Cryptosporidium parvum exacerbated intestinal inflammation in goats by suppressing butyrate-producing bacteria [100]. In lambs, supplementation with sodium butyrate (3 g/kg feed for 28 days) significantly reduced inflammatory responses by decreasing levels of pro-inflammatory cytokines such as TNF-α, IL-1β, and IL-8, and by inhibiting the LPS-induced TLR4/NF-κB signaling pathway at both mRNA and protein levels. Simultaneously, the treatment upregulated the expression of tight junction proteins (e.g., Claudin-3, ZO-1, and Occludin), thereby improving gut barrier function and enhancing the ruminal microbiota [101]. Similarly, Chang et al. [102] revealed that sodium butyrate supplementation significantly increased the pH of both the rumen and cecum, which was associated with the downregulation of GPR41/43 gene expression, inflammatory cytokines (IL-1β, IL-6, and IL-8), and chemokines (CCL5, CXCL13). At the same time, tight junction proteins were upregulated, further strengthening gut barrier integrity. Interestingly, the study also suggested that sodium butyrate induced epigenetic modifications, such as DNA methylation and chromatin compaction, in the promoter regions of GPR41/43 genes, indicating a potential epigenetic mechanism behind the observed changes [102]. In summary, these studies highlight the diverse benefits of sodium butyrate in enhancing intestinal health, growth performance, and the overall physiological well-being of sheep and goats, as illustrated in Table 3.

## 4. Discussion

**Figure 3:**
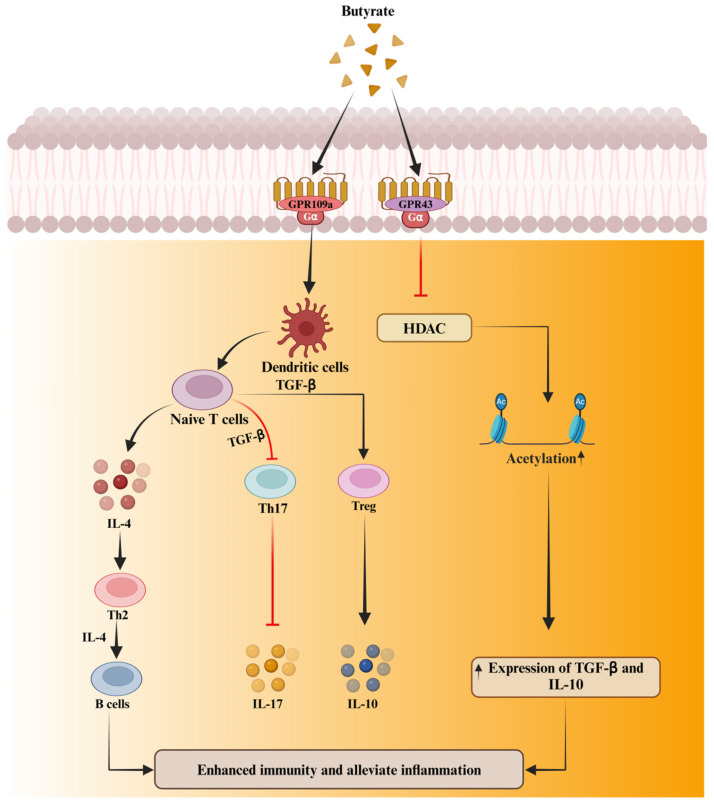
The schematic overview illustrates the role of butyrate in enhancing immunity. Butyrate is crucial for maintaining intestinal immune homeostasis. Butyrate inhibiting histone deacetylases (HDAC) activity by promoting its acetylation. HDAC acetylation significantly upregulates the proliferation and functional efficacy of regulatory T-cells. These cells play a pivotal role in modulating immune responses by attenuating inflammation through the secretion of anti-inflammatory cytokines, including interleu-kin-10 (IL-10) and transforming growth factor-beta (TGF-β) [111,112,113,114].

**Figure 4:**
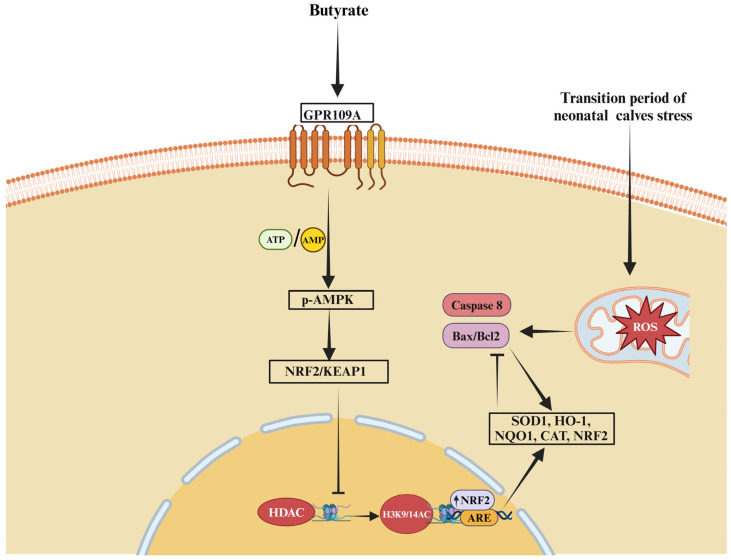
The antioxidant role of butyrate in promoting animal health. Butyrate alleviates stress in high-yielding dairy cows through the GPR109A/AMPK/NRF2 signaling pathway and H3K9/14 acetylation. Furthermore, NRF2 nuclear accumulation and H3K9/14 acetylation enhance antioxidant and anti-apoptotic effects in bovine mammary epithelial cells.

One of the most important functions of butyrate is its ability to regulate inflammatory responses. Its anti-inflammatory effects are mainly achieved by inhibiting NF-κB, a key transcription factor responsible for the expression of pro-inflammatory cytokines such as IL-1β and TNF-α [15,110]. By suppressing NF-κB activation, butyrate effectively reduces the secretion of cytokines, creating a less inflammatory environment. This mechanism is particularly crucial during key developmental stages when organisms are more sensitive to stress and inflammatory conditions [16,17]. Moreover, butyrate’s role as an HDAC inhibitor is central to its anti-inflammatory function [110]. Through HDAC inhibition, butyrate increases histone acetylation, promoting the transcription of anti-inflammatory genes and downregulating pro-inflammatory ones (Figure 2) [19]. Further research indicates that butyrate activates the aryl hydrocarbon receptor (AHR) via MCT1 and sodium-coupled monocarboxylate transporter 1 (SMCT1), contributing to its anti-inflammatory effects. Supporting this, a study showed that HDAC activity increased linearly under higher doses of LPS, while histone H3 acetylation levels significantly decreased [22]. This dual mechanism not only fosters an anti-inflammatory environment but also aids in the differentiation of regulatory T cells (Tregs), which are critical for maintaining immune balance and controlling excessive inflammatory responses [111,112]. Numerous studies have demonstrated that the inhibition of HDAC activity significantly upregulates the proliferation and functional efficacy of regulatory T-cells. These cells play a pivotal role in modulating immune responses by attenuating inflammation through the secretion of anti-inflammatory cytokines, including interleukin-10 (IL-10) and transforming growth factor-beta (TGF-β) [113,114]. Other studies have also reported that butyrate promotes Treg cell differentiation and the secretion of anti-inflammatory mediators such as IL-10 and secretory IgA while inhibiting Th17 cell differentiation, thereby enhancing immune and anti-inflammatory responses (Figure 3) [12,111,115]. Consistently, another study reported that Butyrate treatment significantly downregulated the level of natural killer (NK) cell function during chronic inflammation [116]. NK cells are crucial for defending against infections and tumors by targeting infected or malignant cells. However, in chronic inflammation, overactive or dysregulated NK cells can cause tissue damage by attacking stressed but healthy cells, potentially leading to autoimmune responses. Prolonged activation of NK cells can worsen chronic diseases by triggering tissue inflammation or fibrosis. To prevent this, the immune system must regulate NK cell activity to avoid excessive responses that may harm healthy tissues. In chronic inflammation, dysregulation can lead NK cells to mistakenly target the body’s own tissues, further promoting inflammation and hindering recovery. Research by Shin et al. [117] demonstrated that sodium butyrate (NaB) promotes gene transcription in bovine cells by acetylating histones H3K9 and H3K27. Similarly, another study confirmed that sodium butyrate exerts antioxidant effects through GPR109A, Nrf2, and H3K9/14 acetylation [60]. The study further revealed that butyrate enhances Nrf2 nuclear accumulation and H3K9/14 acetylation via the AMPK signaling pathway, showcasing butyrate’s antioxidant and anti-apoptotic roles through the GPR109A/AMPK/Nrf2 signaling axis. Acetylation of H3K9/14 promotes Nrf2 transcription, thereby boosting the cell’s antioxidant defenses. In addition to its anti-inflammatory and gut-protective functions, butyrate also exhibits significant antioxidant properties (Figure 4). It increases the expression of antioxidant enzymes such as GSH-Px and activates Nrf2, a key regulator of the body’s antioxidant response [71]. By activating Nrf2 and upregulating the expression of antioxidant genes, butyrate helps mitigate oxidative stress, preventing cellular damage and exacerbation of inflammatory processes. Further studies by Correia et al. [75] showed that sodium butyrate enhances ADG and feed conversion efficiency, increases ileal villus height, and reduces the number of Peyer’s patches in the ileum. Sodium butyrate also upregulates the expression of several key genes related to nutrient transport (SGLT1, MCT1), tight junction integrity (OCL, ZO-1), and antioxidant defense (GPX, SOD), all of which are essential for maintaining intestinal function and integrity [75].

## 5. Conclusions

In conclusion, butyrate supplementation has emerged as a promising strategy for enhancing intestinal health and growth performance in livestock. Its multifaceted benefits include improving gut barrier integrity, modulating immune responses, optimizing microbial diversity, and reducing inflammation and oxidative stress. Butyrate’s role in gene expression and epigenetic regulation further underscores its potential to promote tissue development and metabolic efficiency. Studies across calves, piglets, sheep, and goats consistently demonstrate its ability to enhance average daily gain, feed efficiency, and overall health, particularly during critical developmental stages like weaning. Future opportunities in this area include optimizing butyrate supplementation protocols to maximize benefits across various livestock species, developmental stages, and environmental conditions. Research should focus on precise dosing, long-term health impacts, and the mechanisms of butyrate’s interaction with the gut microbiome and immune system. Exploring synergistic effects with other feed additives and their potential in disease mitigation could lead to new therapeutic applications. Future studies should also explore the potential of butyrate as an alternative to antibiotics as a growth promoter, emphasizing its role in mitigating the issue of antibiotic resistance. By tailoring strategies to species-specific needs, butyrate can be optimized as a valuable dietary additive, enhancing livestock productivity and welfare sustainably.

## Figures and Tables

**Table 1:** Summary of research on butyrate’s role in enhancing intestinal health and growth performance in calves.

Biological Effect of Butyrate Treatment	Experimental Model	References
Sodium butyrate (1 mM for 15 h), followed by LPS treatment (5 μg/mL for 3 h), attenuated LPS-induced inflammatory responses by downregulating the expression of IL-6, IL-1β, COX-2, NLRP3, and iNOS, and by inhibiting the phosphorylation of NF-κB, IκB, and p65 induced by LPS in bovine macrophages.	Bovine Macrophages	[59]
Sodium butyrate (5 mM, 18 h) followed by LPS treatment (1 µg/mL, 6 h) inhibited the LPS-induced upregulation of TNF-α, IL6, and IL1B, and suppressed NF-κB p65 phosphorylation (5 µg/mL) in BMECs.	BMECs	[58]
Sodium butyrate (2 mM for 12 h) enhanced the antioxidant response in H_2_O_2_ treated (600 µM for 12 h) BMECs by activating G protein-coupled receptor 109A (GPR109A) and nuclear factor erythroid 2-related factor 2 (Nrf2), while promoting H3K9/14 acetylation. This treatment also facilitated Nrf2 nuclear accumulation through the AMPK signaling pathway, thereby promoting antioxidant and anti-apoptotic activities via the GPR109A/AMPK/Nrf2 signaling axis. Additionally, increased H3K9/14 acetylation further stimulated Nrf2 transcription, contributing to an enhanced antioxidant capacity in BMECs	BMECs	[60]
LPS (100 EU/mL) and LPS (100 EU/mL) plus sodium butyrate (10 mmol/L) treatments for 48 h increased the expression of lactation-related genes, including ACACA and ribosomal protein S6 kinase 1 (S6K1), which had been downregulated by LPS treatment in MAC-T cells. Additionally, sodium butyrate treatment suppressed HDAC activity, leading to a decline in histone H3 acetylation.	MAC-T cells	[22]
Sodium butyrate (5 mM) treatment for 2 h prior to LPS (1 µg/mL) inhibited HDACs, leading to enhanced acetylation of H3K9/14, H3K18, and H3K27 in MAC-Ts. However, despite the changes in HDAC activity and histone acetylation, sodium butyrate did not significantly reduce overall inflammatory gene expression.	MAC-Ts	[61]
Pretreatment with 0.5 mmol/L sodium butyrate for 18 h followed by a 6 h LPS challenge (4 μg/mL) in bovine hepatocytes showed that LPS exposure increased TNF-α and IL-6 production, but sodium butyrate pretreatment significantly reduced these levels in bovine hepatocytes. Sodium butyrate also inhibited the expression and nuclear translocation of phospho-p65 and phospho-IκBα, key proteins involved in inflammatory signaling stimulated by LPS. Additionally, genes and proteins associated with fatty acid metabolism, such as SREBP1c, SCD1, and DGAT1, were upregulated in cells pretreated with sodium butyrate. The ratios of phospho-AMPKα to AMPKα and phospho-ACCα to ACCα were significantly lower in the sodium butyrate-pretreated group, indicating altered fatty acid oxidation. Furthermore, sodium butyrate reversed the histone H3 deacetylation induced by LPS, suggesting a protective epigenetic effect.	Bovine Hepatocytes	[62]
The combination of milk replacer (MR) with 0.24% butyrate supplementation has shown greater MR intake and body weight in calves, exhibiting higher plasma IGF-I and IGFBP-3 levels, and lower IGFBP-2 levels. After weaning, plasma IGF-I and IGFBP-4 levels decreased in calves, while IGFBP-2 levels increased. At day 50, the combination of sodium butyrate and milk replacers resulted in higher mRNA levels of *GHR1A* and *IGF1*, along with lower levels of *IGFBP2*.	Dairy calves	[63]
Calves fed MR with 0.24% butyrate supplementation ad libitum had higher MR intake and body weight, with butyrate decreasing the sucking rate but increasing MR intake per meal. All calves showed increased immunoglobulin levels after colostrum intake. Body temperature was higher in ad libitum-fed calves during the first two weeks, suggesting better welfare due to fewer signs of hunger. However, neither feeding strategy nor butyrate supplementation consistently impacted health or immune status.	Dairy calves	[64]
Sodium butyrate, included in milk replacer at 4 g/kg of dry matter (DM) from days 7 to 56 of life, improved the concentrations of propionate, acetate, and total SCFAs in the colon of calves. This supplementation decreased the cecal abundance of butyrate-producing bacteria, *Butyrivibrio* and *Shuttleworthia* while enhancing the level of the propionate producer *Phascolarctobacterium*. Concurrently, it decreased the cecal concentration of *Mogibacterium*, which was associated with impaired gut health.	Dairy calves	[65]
Sodium butyrate supplementation along with a high-starch diet for a 21-day period significantly improved plasma GLP-2 concentration, increased n-butyrate concentration in the ruminal fluid, and tended to enhance the digestibility of dry matter and organic matter in Holstein cows.	Holstein cows	[66]
Tributyrin supplementation in milk replacers has been shown to reduce the occurrence of diarrhea in preweaning Holstein calves. This dietary intervention enhances gut health by promoting the development of the intestinal epithelium and improving gut barrier function, which in turn helps mitigate gastrointestinal disturbances commonly observed in young calves. The addition of tributyrin plays a crucial role in maintaining intestinal integrity and supporting the overall health and well-being of preweaning Holstein calves.	Holstein’s calves	[67]
Sodium butyrate supplementation (4.4 g/day for 6 weeks) has been found to promote the ruminal microbiota, enhancing microbial diversity and stability within the rumen. This intervention positively influences the composition and activity of ruminal microorganisms, supporting improved fermentation processes and nutrient utilization. By fostering a more balanced microbial environment, sodium butyrate contributes to better overall ruminal health and function, which can enhance the digestive efficiency and performance of Holstein calves.	Holstein’s calves	[68]

**Table 2:** Summary of research on butyrate’s role in enhancing intestinal health and growth performance in pigs.

Biological Effect of Butyrate Treatment	Experimental Model	References
Sodium butyrate (3.00 g/kg coated butyrate for 21 days), followed by LPS treatment (100 μg/kg body weight), significantly improved intestinal health by enhancing villus height, improving the villus height to crypt depth ratios, and reducing tissue damage in piglets. Butyrate also reversed the LPS-induced activation of inflammatory pathways, including NF-κB p65 and PPARα, decreased pro-inflammatory cytokines such as IL-1β, IL-6, TNF-α, and TLR4, and promoted anti-inflammatory responses by increasing IL-10 and IL-13 levels. Additionally, butyrate modulated the piglets’ gut microbiota by increasing beneficial bacteria, including *Firmicutes*, *Bacteroidetes*, *Clostridiaceae*, *Lactobacillus*, and *Prevotella*, while reducing harmful bacteria like *Proteobacteria*, *Enterobacteriaceae*, and *Escherichia/Shigella.* It also reduced apoptosis by regulating mitochondrial pathways and enhanced enterocyte energy metabolism.	Piglets	[70]
The inclusion of 3 kg of protected sodium butyrate/ton of feed enhanced the pig intestinal microbiota, promoting the growth of beneficial families such as *Prevotellaceae*, *Lachnospiraceae*, *Peptostreptococcaceae*, *Peptococcaceae*, and *Terrisporobacter.* This supplementation also promoted *Deinococcus-Thermus*, a group associated with the production of carotenoids that possess antioxidant, anti-apoptotic, and anti-inflammatory properties. Furthermore, the increased presence of *Clostridium butyricum* was linked to overall positive gut effects in weanling pigs, including improvements in villus height, body weight, and a reduction in diarrhea.	Piglets	[81]
The inclusion of 3 kg of protected sodium butyrate/ton of feed reduced the clinical and histological severity of colitis while decreasing pro-inflammatory cytokines such as IL-1β, IL-6, IL-8, and TNF-α in piglets. It promoted the growth of beneficial gut bacteria, including Faecalibacterium and Lactobacillus, and enhanced the tricarboxylic acid (TCA) cycle in colonocytes, thereby improving metabolism in the gut-liver axis. Additionally, the supplementation increased butyric acid levels in the colon and portal vein, facilitated the utilization of amino acids and vitamin B, and reversed LPS-induced fatty acid synthesis. Furthermore, it enhanced the production of anti-inflammatory cytokines, including IL-10 and TGF-β, while suppressing inflammatory mediators such as hypoxia-inducible factor 1α.	Piglets	[82]
The supplementation of a basal diet with 0.2% sodium butyrate demonstrated significant improvements in growth performance, as evidenced by enhanced final body weight, daily weight gain, and daily feed intake, alongside better feed efficiency and increased carcass weight in piglets. Additionally, it positively influenced the gut microbiota by promoting the abundance of *Bacteroidetes* while reducing the levels of *Firmicutes* and *Proteobacteria* in the caecal environment.	Piglets	[83]
The addition of 0.2% sodium butyrate in a basal diet counteracted the detrimental effects of 4 mg/kg deoxynivalenol (DON) exposure, which typically led to decreased performance, impaired intestinal barrier integrity, reduced expression of host defense peptides (HDPs), and disrupted gut microbiota in pigs. Sodium butyrate supplementation not only mitigated these adverse outcomes but also improved liver health and reduced levels of harmful enzymes. In vitro studies further demonstrated its protective role against DON-induced damage. Mechanistically, sodium butyrate activated the NOD2/caspase-12 pathway, subsequently enhancing HDP expression, which played a critical role in preventing intestinal barrier dysfunction caused by DON.	piglets	[84]
The supplementation of coated sodium butyrate at a dosage of 450 mg/kg significantly enhanced ADG, improved the *Lactobacilli* to *E. coli* ratio in the jejunum, and effectively reduced the incidence of post-weaning diarrhea and intestinal permeability in piglets.	Piglets	[85]
The supplementation of 5 g sodium butyrate per kilogram of feed during the first three weeks significantly enhanced growth performance in piglets, as evidenced by increased final body weight, ADG, and feed efficiency. Additionally, it improved dry matter digestibility, reduced coliform counts, and promoted the growth of lactic acid bacteria in the intestine. Furthermore, fecal gas emissions, particularly hydrogen sulfide (H_2_S), were reduced, and villi length in the small intestine showed notable improvement, contributing to overall intestinal health and functionality.	Piglets	[86]
The administration of 450 mg of sodium butyrate/kg of feed over a two-week period significantly enhanced growth performance and improved intestinal structure in piglets, as indicated by increased villus height and strengthened intestinal barrier function. Additionally, it reduced mast cell degranulation and decreased levels of inflammatory mediators, including histamine, tryptase, TNF-α, and IL-6, in the intestines. The suppression of the JNK signaling pathway activation further demonstrated that sodium butyrate protects intestinal integrity by inhibiting mast cell activation and inflammation through this mechanism, highlighting its potential role in promoting intestinal health and reducing inflammatory responses.	Piglets	[72]
The intravenous administration of 40 mg of sodium butyrate/kg of body weight over seven days resulted in the upregulation of *MCT1* in the colon and a reduction in the expression of *HDAC1* and pro-inflammatory cytokine genes, including *IL-6*, *IL-18*, *IL-12p40*, and *TNF-α.* This treatment also bolstered anti-inflammatory responses and enhanced key markers of intestinal development in piglets, such as ZO-1, occludin, and epidermal growth factor (EGF), demonstrating its potential to support intestinal integrity and modulate inflammatory pathways effectively.	Piglets	[87]
The supplementation of 2000 mg of sodium butyrate/ kg of feed for 21 days significantly reduced diarrhea incidence and enhanced intestinal barrier function in piglets by upregulating key tight junction proteins, including Claudin-3, Occludin, and ZO-1, in the piglet colon. These beneficial effects were mediated through the activation of the GPR109A and the Akt signaling pathway. In vitro studies further supported these findings, demonstrating that blocking either GPR109A or Akt led to a reduction in Claudin-3 expression, confirming that sodium butyrate promotes intestinal integrity and barrier function through these specific molecular mechanisms.	Piglets	[88]
The inclusion of 1 g sodium butyrate/kg in the basal diet from day 85 of gestation through day 22 of lactation resulted in a shortened weaning-to-estrus interval and a reduced incidence of diarrhea in piglets. It also enhanced the nutritional and immunological quality of colostrum, as evidenced by increased concentrations of fat, protein, IgA, IgG, and IgM. Furthermore, sodium butyrate upregulated mRNA expression of key intestinal health markers, including claudin-1, zona occludens 1, and interleukin-10 while downregulating *TLR4* expression. Additionally, it influenced microbial diversity by reducing the abundance of *Firmicutes*, *Actinobacteria*, and *Synergistetes*, highlighting its role in improving reproductive performance, colostrum quality, and intestinal health.	Piglets	[89]

**Table 3:** Summary of research on butyrate’s role in enhancing intestinal health and growth performance in sheep and goats.

Biological Effect of Butyrate Treatment	Experimental Model	References
The administration of coated sodium butyrate at a dosage of 3 g/kg over a 21-day period significantly enhanced the colonic microbiota in weaned lambs, promoting the growth of beneficial bacterial families such as *Lachnospiraceae*, *Verrucomicrobiota*, *Akkermansia*, *Roseburia*, and *Sinobacteraceae*. This intervention also improved the ADG and ADFI of the lambs. Additionally, it enhanced the antioxidant capacity, as evidenced by increased levels of T-AOC, T-SOD, GSH-Px, and CAT, while reducing MDA levels, thereby mitigating weaning stress.	Lamb	[99]
The inclusion of 6.25 g of calcium butyrate/kg of diet dry matter significantly enhanced feed intake and ADG while improving liver health in lamb, as indicated by reduced levels of alanine aminotransferase (ALT) and aspartate aminotransferase (AST).	Lamb	[103]
Sodium butyrate demonstrated the ability to enhance antimicrobial responses in goat mammary gland cells by significantly increasing the concentrations of β-defensin-1 and S100A7 in milk, thereby bolstering the innate immune defense mechanisms.	Goat MECs	[104]
The supplementation of tributyrin at 4.0 g/kg of DM significantly improved ADG and increased dry matter intake (DMI), while enhancing nutrient retention by reducing nitrogen, calcium, and phosphorus losses through feces and urine in lamb. It also promoted gastrointestinal development, evidenced by increased papillae length and width in the dorsal and ventral sacs and caudodorsal blind sac, as well as greater duodenal and ileal thickness and increased villus height in the duodenum, ileum, jejunum, and cecum. Additionally, reduced crypt depths in the duodenum and cecum suggested improved epithelial turnover and absorption efficiency. Tributyrin further enhanced rumen fermentation, as indicated by a significant decrease in rumen pH and an increase in total volatile fatty acid (VFA) concentration, while enriching the rumen microbial community by significantly boosting populations of *Clostridium*, *Butyrivibrio*, *Streptococcus*, *Prevotella*, *Ruminobacter*, and *Fibrobacter*, which are key bacteria associated with VFA production and fiber digestion.	Lambs	[105]
Sodium butyrate effectively mitigated oxidative stress induced by grain-induced sub-acute ruminal acidosis (SARA) in dairy goats by increasing mean ruminal pH and reducing LPS levels in the ruminal, portal, and hepatic regions. It enhanced the mRNA expression of antioxidant-related genes (*SOD1*, *SOD2*, *SOD3*, *GPX1*, and *CAT*), elevated TSOD and CAT enzyme activities, improved T-AOC, and reduced MDA levels in both the liver and plasma. Additionally, sodium butyrate upregulated the mRNA of expression of *UGT1A1*, *NQO1*, *MGST3*, and *Nrf2*, along with increasing total Nrf2 protein levels in goats fed a high-grain diet supplemented with sodium butyrate (HG + NaB).	Goat	[106]
The inclusion of 1% sodium butyrate effectively reversed damage to the rumen epithelium tight junction by inhibiting the protein kinase C (PKC) and MAPK signaling pathways, thereby providing protective effects on the rumen epithelium during subacute rumen acidosis induced by a high-concentrate diet.	Goat	[107]
The supplementation of 3 g of sodium butyrate/kg of starter DM over 73 days significantly improved starter intake and increased weaning weight in lambs. While forage provision alone did not affect intake or weight gain, the combination of sodium butyrate and forage demonstrated a positive interaction, enhancing lamb weight gain. Lambs in the forage-sodium butyrate group exhibited the greatest wither height and a larger body barrel. Additionally, this treatment led to increased concentrations of SCFAs and butyrate, further supporting its beneficial effects on growth and development.	Lamb	[90]
Oral infusion of sodium butyrate at a dose of 0.36 g/kg body weight from 10 to 49 days of age significantly increased ADFI, ADG, and BW of lambs at 5 and 6 weeks. The treatment resulted in higher concentrations of acetate, butyrate, and total volatile fatty acids (VFA) in rumen fluid, along with elevated levels of β-hydroxybutyrate (BHBA), insulin-like growth factor-1 (IGF-1), and insulin in plasma. Sodium butyrate promoted rumen papillae growth, as evidenced by increased rumen weight, papillae length, width, surface area, and thickness of the stratum corneum and epithelium. It upregulated the mRNA expression of genes related to cell cycle regulation (*cyclin A2*, *cyclin D1*, *CDK6*) and downregulated pro-apoptotic genes (*caspase-3*, *Bax*). Additionally, sodium butyrate elevated the expression of IGF-1 signaling genes (*IGF-1R*, *IGFBP-5*) while downregulating *IGFBP-3*. It also increased the mRNA expression of the VFA transporter *MCT1* gene and decreased the expression of genes involved in ion transport (*NHE2*) and fatty acid synthesis (*HMGCS2*, *HMGCL*).	Lamb	[108]
Intraruminal infusion of sodium butyrate at a dose of 0.3 g/kg of body weight, administered in 50 mL of 0.1 mol/L potassium phosphate buffer for 28 days, was associated with elevated ruminal butyrate concentration, larger papillae, and a higher number of cell layers in the epithelial strata of lamb. The treatment increased the mRNA expression of cell cycle regulators (*cyclin A*, *cyclin D1*, *cyclin E1*, *CDK2*, *CDK4*, and *CDK6*) and upregulated apoptosis-linked genes (*caspase 3*, *caspase 9*, and *Bax*).	Lamb	[109]

## Data Availability

Not applicable.
